# A Novel Quinazoline Derivative Prevents and Treats Arsenic-Induced Liver Injury by Regulating the Expression of RecQ Family Helicase

**DOI:** 10.3390/ijms242115521

**Published:** 2023-10-24

**Authors:** Heping Yang, Min Mo, Langlang Yang, Jia Yu, Jiao Li, Sha Cheng, Baofei Sun, Bixue Xu, Aihua Zhang, Heng Luo

**Affiliations:** 1 The Key Laboratory of Environmental Pollution Monitoring and Disease Control, Ministry of Education, Guizhou Medical University, Guiyang 550025, Chinamorrim2022@163.com (M.M.); sunbaofei@gmc.edu.cn (B.S.); 2 State Key Laboratory of Functions and Applications of Medicinal Plants, Guizhou Medical University, Guiyang 550014, China; yujia@gzcnp.cn (J.Y.); chengsha@gzcnp.cn (S.C.); bixue.xu@gzcnp.cn (B.X.); 3 Natural Products Research Center of Guizhou Province, Guizhou Medical University, Guiyang 550014, China

**Keywords:** quinazoline derivative, sodium arsenite, liver injury, RecQ helicase, prevention and treatment effects

## Abstract

Arsenic is a carcinogenic metalloid toxicant widely found in the natural environment. Acute or prolonged exposure to arsenic causes a series of damages to the organs, mainly the liver, such as hepatomegaly, liver fibrosis, cirrhosis, and even hepatocellular carcinoma. Therefore, it is imperative to seek drugs to prevent arsenic-induced liver injury. Quinazolines are a class of nitrogen heterocyclic compounds with biological and pharmacological effects in vivo and in vitro. This study was designed to investigate the ameliorating effects of quinazoline derivatives on arsenic-induced liver injury and its molecular mechanism. We investigated the mechanism of the quinazoline derivative KZL-047 in preventing and ameliorating arsenic-induced liver injury in vitro by cell cycle and apoptosis. We performed real-time fluorescence quantitative polymerase chain reaction (qPCR) and Western blotting combined with molecular docking. In vivo, the experiments were performed to investigate the mechanism of KZL-047 in preventing and ameliorating arsenic-induced liver injury using arsenic-infected mice. Physiological and biochemical indices of liver function in mouse serum were measured, histopathological changes in liver tissue were observed, and immunohistochemical staining was used to detect changes in the expression of RecQ-family helicases in mouse liver tissue. The results of in vitro experiments showed that sodium arsenite (SA) inhibited the proliferation of L-02 cells, induced apoptosis, blocked the cell cycle at the G1 phase, and decreased the expression of RecQ family helicase; after KZL-047 treatment in arsenic-induced L-02 cells, the expression of RecQ family helicase was upregulated, and the apoptosis rate was slowed, leading to the restoration of the cell viability level. KZL-047 inhibited arsenic-induced oxidative stress, alleviated oxidative damage and lipid peroxidation in vivo, and ameliorated arsenic toxicity-induced liver injury. KZL-047 restored the expression of RecQ family helicase proteins, which is consistent with the results of in vitro studies. In summary, KZL-047 can be considered a potential candidate for the treatment of arsenic-induced liver injury.

## 1. Introduction

Arsenic is a highly toxic metalloid element, widely distributed in both inorganic and organic forms in soil, water, air, plants, and microorganisms in nature [1]. Therefore, arsenic easily enters human and animal bodies through food, water, and industrial pollutants [2]. Epidemiological studies have shown that the liver is one of the important target organs for arsenic toxicity [3], and chronic arsenic exposure can lead to liver fibrosis, cirrhosis, and liver cancer [4,5]. According to statistics, presently, more than 200 million individuals worldwide are faced with excessive arsenic content in drinking water, and more than three million individuals in China are exposed to arsenite-contaminated water. Chronic arsenic poisoning has become an environmental problem of concern to the world today [6]. Therefore, it is of great significance to study the prevention and treatment of arsenic poisoning.

At present, the mechanism underlying liver injury, liver fibrosis, liver cirrhosis, and liver cancer caused by arsenic accumulation in the liver is not well defined [7]. Studies have shown that the mechanism of arsenic pathogenesis, including the biological metabolism of arsenic is very complex; arsenic induces oxidative stress, influences cell apoptosis, disrupts the cell cycle, inhibits DNA damage repair, induces genetic damage, and changes epigenetic patterns [8,9]. Oxidative stress and DNA damage repair are widely accepted and studied mechanisms of arsenic toxicity. Further, apoptosis induced by reactive oxygen species (ROS) accumulation may play an important role in arsenic-induced poisoning mechanisms [10]. DNA damage repair is another major mechanism of arsenic toxicity. Patients with drinking water-type arsenic poisoning have been found to have high levels of DNA damage in peripheral lymphocytes or exfoliated cells, with increased frequencies of sister chromatid exchange and micronucleus and chromosomal aberrations [11]. Helicases are enzymes that untie the nucleotide double chain. RecQ helicase is a class of proteins involved in maintaining the stability of the genome and is highly conservative in bacteria and even human beings. These helicases play an important role in maintaining the genetic stability of various organisms and also play a key role in the metabolic process of DNA replication, repair, and recombination [12,13]. Studies have found that there are at least five RecQ helicases in human cells, including BLM, WRN, RecQL1, RecQL4, and RecQL5.

Quinazoline is a heterocyclic compound comprising a benzene ring fused with a pyrimidine ring, and quinazoline derivatives are a class of important heterocyclic compounds commonly found in nature, with excellent biological activities and pharmacological effects, such as antihepatitis B, antitumor, antibacterial, anti-inflammatory, antituberculosis, insecticidal, and herbicidal effects [14,15]. Quinazoline derivatives have the advantages of low toxicity, high efficiency, unique mode of action, and easy modification, and have become an important topic in the field of pharmaceutical chemistry and pharmacology [16,17]. However, the protective effects of quinazoline derivatives against liver injury caused by sodium arsenite (SA) have not been reported.

Our studies have shown that L-02 cell viability decreased after arsenic exposure; arsenic-induced apoptosis of cells and the degree of cell damage worsened with an increasing dose of arsenic. Further, mRNA and protein expression of RecQL1, WRN, RecQL4, and RecQL5 genes decreased. Based on this, we investigated the effects of KZL-047 on the proliferation, apoptosis, cell cycle, and molecular mechanisms of arsenic-induced liver injury, and the results showed that KZL-047 could upregulate the expression of the RecQ family helicase gene in arsenic-stained L-02 cells, slow cell apoptosis, and restore cell viability. These findings suggested KZL-047 as a potential candidate drug for the treatment of arsenic-induced liver injury.

## 2. Results

### 2.1. Effect of KZL-047 on the Growth of L-02 Cells

#### 2.1.1. Effect of Different Concentrations of KZL-047 on the Proliferation of L-02 Cells

**Figure 1:**
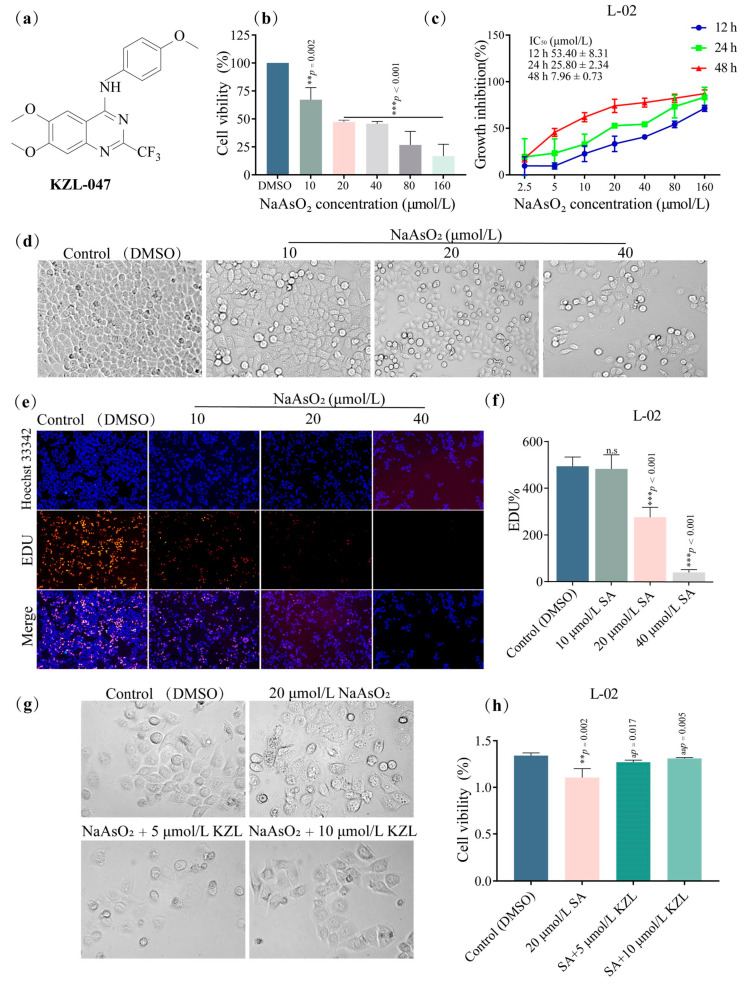
Effect of NaAsO_2_ (SA) on L-02 cell growth at various concentrations: (**a**) chemical structure of KZL-047; (**b**) preliminary viability screening for L-02 cells after exposure to SA; (**c**) growth inhibition curve for L-02 cells treated with SA for 12, 24, and 48 h, as determined by the MTT assay; (**d**) morphological changes in L-02 cells treated with SA for 24 h as observed under a microscope; (**e**) determination of the inhibitory effect of SA on L-02 cells by the EdU method as observed under an inverted fluorescence microscope; (**f**) Image J software (V1.8.0.112, NIH, Bethesda, MD USA) quantitative statistics analysis of EdU staining pictures; (**g**) morphological map showing the effect of KZL-047 on SA-induced damage to L-02 cells; (**h**) statistical chart showing the effect of KZL-047 on the viability of SA-treated L-02 cells. The abovementioned images are all magnified at 100×. Data results are expressed as mean ± SD of three independent experiments. ** *p* < 0.01, *** *p* < 0.001 compared with the control group; ^a^ *p* < 0.05, ^aa^ *p* < 0.01 compared with the model group (*n* = 3).

Growth analysis of L-02 cells after treatment showed that the viability of L-02 cells decreased with increasing SA concentrations (0, 10, 20, 40, 80, and 160 µmol/L) (Figure 1b). The IC_50_ values of L-02 cells following treatment with SA at 12, 24, and 48 h were 53.40 ± 8.31, 25.80 ± 2.34, and 7.96 ± 0.73 µmol/L (Figure 1c), indicating a time-dependent inhibitory effect of SA on L-02 cells. Microscopic observation (Figure 1d) showed that the number of cells decreased, cell morphology changed from a regular spindle shape to a round shape, and cell gaps became larger with increasing concentration and treatment time. In the follow-up intervention experiment, an exposure dose of 20 µmol/L was selected for 24 h. The inhibitory effect of SA on L-02 cell proliferation was further tested using an EdU test kit. The results showed that (Figure 1e) the number of red-stained cells decreased as SA concentration increased compared with the control group (Figure 1f). The difference in cell changes between 20 and 40 µmol/L was statistically significant (*p* < 0.001).

In the intervention experiment, the cells were divided into a control group (DMSO), model group (20 µmol/L SA), the intervention groups (SA+5 µmol/L KZL-047 and SA+10 µmol/L KZL-047), with continuous treatment of 24 h. The cell morphology was observed through a microscope (Figure 1g). Compared with the control group, the model group showed a decreased number of cells, irregular cell morphology, and rough cell edges. After KZL-047 treatment, the damaged cells were significantly improved, their viability increased (*p* < 0.05), and the degree of cell inhibition was significantly reduced (Figure 1h). This result suggests that KZL-047 alleviated the inhibitory effect of SA on L-02 cells.

#### 2.1.2. Effect of Different Concentrations of KZL-047 on Apoptosis of L-02 Cells

**Figure 2:**
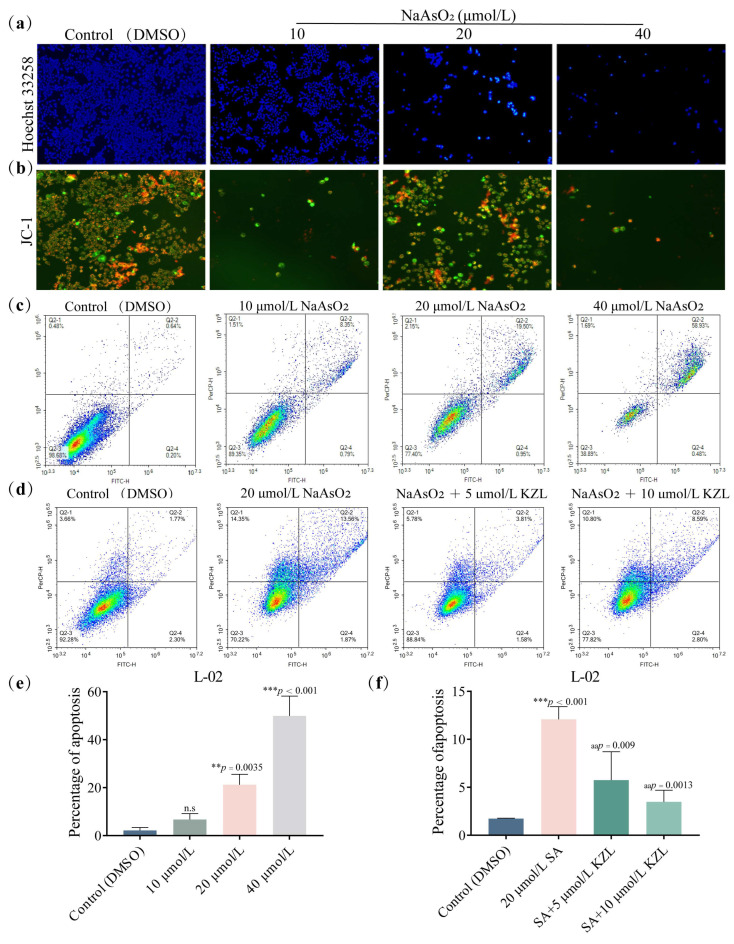
Effect of KZL-047 on L-02 cell apoptosis at different concentrations: (**a**) apoptosis of L-02 cells induced by SA via Hoechst 33258 staining; (**b**) JC-1 staining to observe the effect of SA on the mitochondrial membrane potential of L-02 cells; (**c**) detection of apoptosis of L-02 cells induced by SA via flow cytometry; (**d**) observation of the percentage change in SA-induced apoptosis of L-02 cells following KZL-047 treatment by flow cytometry; (**e**) statistical chart showing the percentage of apoptosis of L-02 cells induced by SA via flow cytometry; (**f**) statistical chart showing the percentage of apoptosis of L-02 cells induced by SA following KZL-047 treatment. The above images are all magnified at 100×.Data results are expressed as mean ± SD of three independent experiments. ** *p* < 0.01, *** *p* < 0.001 compared with the control group; ^aa^ *p* < 0.01 compared with the model group (*n* = 3).

Flow cytometry analysis showed that after continuous treatment of L-02 cells with different concentrations of NaAsO_2_ (SA) for 24 h and an increasing SA concentration, the number of L-02 cells gradually decreased, chromatin agglutinated, the intercellular space became larger, and the nucleus became smaller; the cells in the high-concentration group produced a white bright light (Figure 2a).

Changes in cell membrane potential are a sign of changes in mitochondrial energy metabolism and an important step in the occurrence of apoptosis. JC-1 acts as a fluorescence probe that generates red fluorescence when the mitochondrial membrane potential is high and green fluorescence when the membrane potential is low. To determine whether SA is involved in changes in cell membrane potential while inducing L-02 cell apoptosis, JC-1 was used to stain the cells treated with SA for 24 h, and the changes in cell morphology and color were observed under a fluorescent inverted microscope. The results showed that with an increase in SA concentration, the number of red-stained cells decreased whereas the number of green-stained cells increased, and the membrane potential decreased. This indicated SA-induced apoptosis of L-02 cells in a dose-dependent manner with a decrease in membrane potential (Figure 2b).

To further determine the effect of SA on L-02 cell apoptosis, flow cytometry was performed. L-02 cells were continuously treated with different concentrations of SA for 24 h and were then collected. L-02 cells were stained by the Annexin V-FITC/PI double staining method, followed by apoptosis detection via flow cytometry (Figure 2c). As shown in Figure 2, the apoptosis rate of L-02 cells increased with an increase in SA concentration (Figure 2e). The apoptosis rate in the high-concentration group (40 µmol/L NaAsO_2_) was 32.22% (*p* < 0.05). All the abovementioned results indicate that SA induced apoptosis of L-02 cells.

Flow cytometry was performed to determine the change in percentage in SA-induced apoptosis of L-02 cells following KZL-047 treatment (Figure 2d). The results showed that SA at 20 μmol/L induced apoptosis of L-02 cells with a statistically significant difference (*p* < 0.01) compared with the control group. Compared with the model group, the intervention groups with 5 and 10 μmol/L KZL-047 showed a decrease in the apoptosis rate in a dose-dependent manner. The intervention group with KZL-047 dose of 10 μmol/L cells showed an apoptosis rate that decreased to 3.81% (*p* < 0.01), which was close to that of the control group (DMSO) (Figure 2f). This result suggests that KZL-047 alleviates the SA-induced apoptosis of L-02 cells.

#### 2.1.3. Effect of Different Concentrations of KZL-047 on the Cycle of L-02 Cells

**Figure 3:**
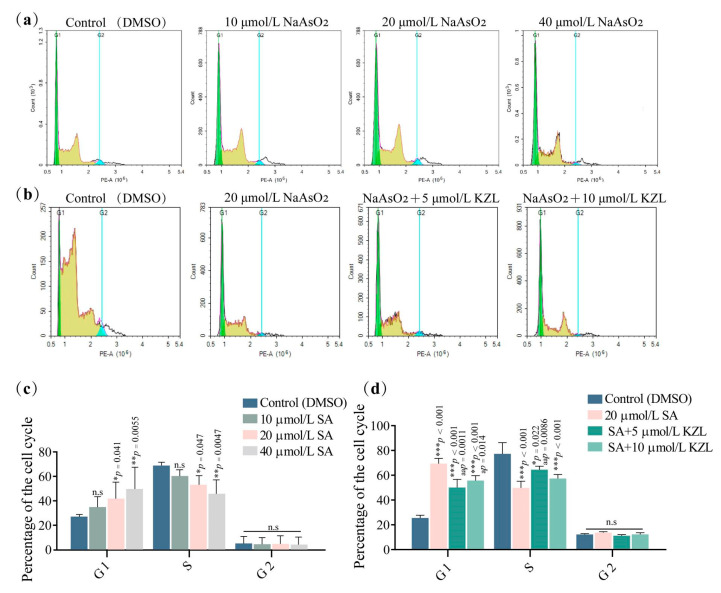
Effect of KZL-047 on L-02 cell cycle at different concentrations: (**a**) observation of changes in L-02 cell cycle following SA treatment via flow cytometry; (**b**) flow cytometry observation of changes in L-02 cell cycle following treatment with SA and KZL-047; (**c**) statistical chart showing the proportion of L-02 cells in each phase between groups following SA treatment; (**d**) statistical chart showing the proportion of L-02 cells in each phase between groups following treatment with KZL-047 and SA. Data are expressed as the mean ± SD of three independent experiments. * *p* < 0.05, ** *p* < 0.01, *** *p* < 0.01 compared with the control group, ^a^ *p* < 0.05, ^aa^ *p* < 0.01 compared with the model group (*n* = 3).

Flow cytometry was performed to detect the effect of SA on the proportion of L-02 cell cycle phases with the collected cells stained with PI and RNase A enzymes. The experimental results were as follows (Figure 3a,c). Compared with the control, treatment with SA doses of 10 and 20 μmol/L resulted in an increase in the area percentage of the G1 phase (*p* < 0.01) and a decrease in the area percentage of the S phase (*p* < 0.05) in a dose-dependent manner; the area percentage of the G2 phase did not significantly change.

Flow cytometry was performed to observe the changes induced in the L-02 cell cycle by KZL-047 following SA treatment. The cell suspensions were divided into a control group (DMSO), model group (20 µmol/L SA), and intervention groups (SA + 5 µmol/L KZL-047 and SA + 10 µmol/L KZL-047); each group was treated for 24 h. The results (Figure 3b,d) indicated that the model group first showed an increase in the G1 phase compared to the control group, with a decrease in the proportion of G1 phase and an increase in S phase after KZL-047 intervention treatment. No significant differences were observed between the groups in the G2 phase. Compared with the model group, KZL-047 alleviated the SA-induced G1 phase block in L-02 cells, which was statistically different (a*p* < 0.05).

### 2.2. Effect of KZL-047 on the RecQ Helicase Expression of L-02 Cells

**Figure 4:**
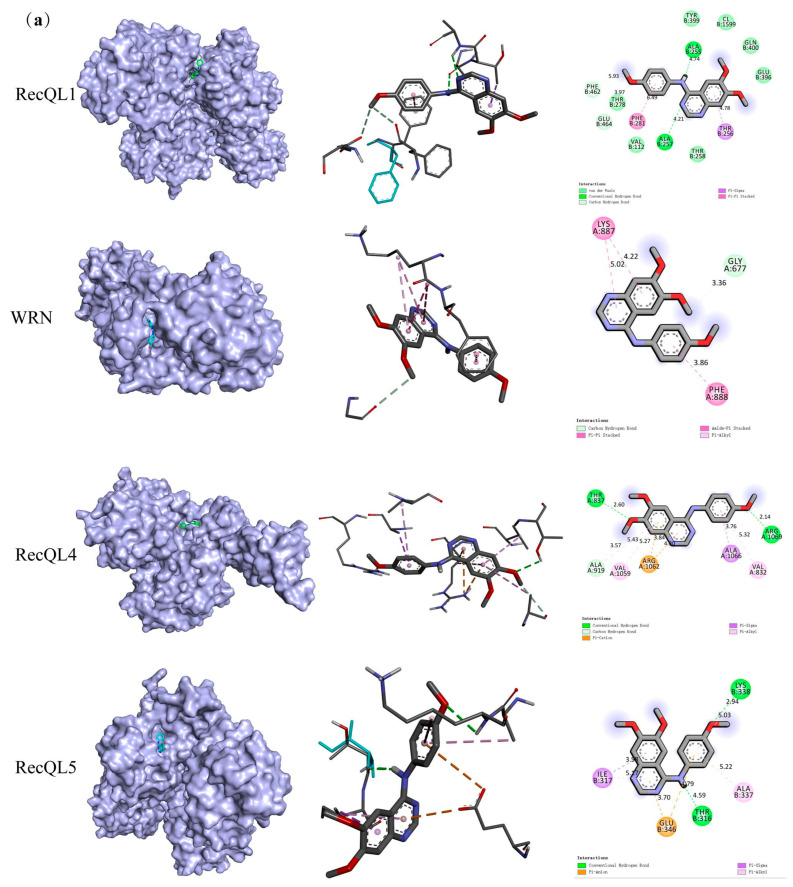

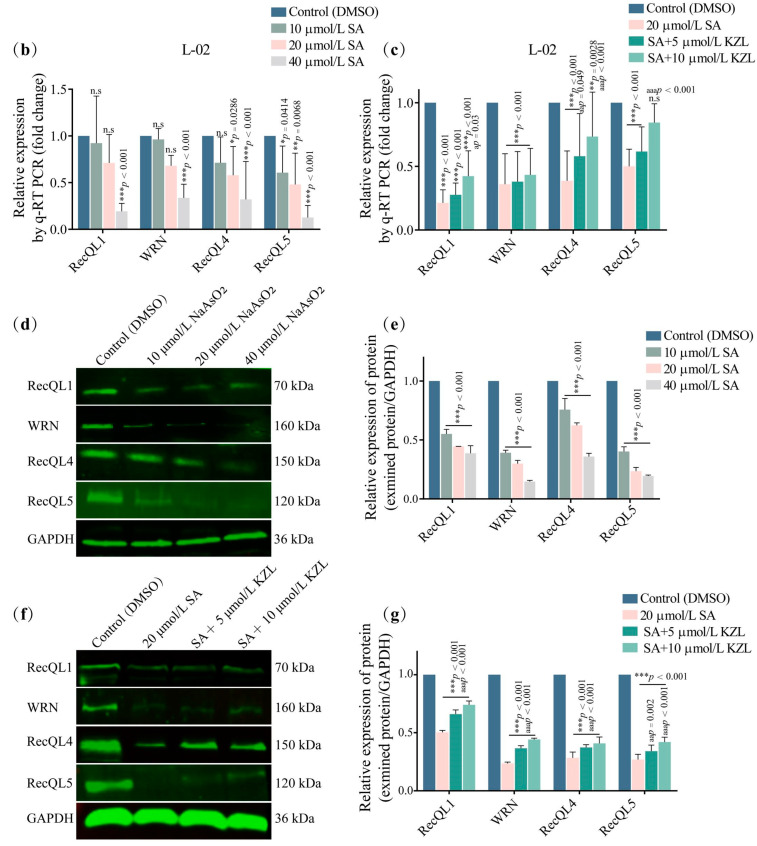
Effect of KZL-047 on the RecQ helicase expression levels in L-02 cells: (**a**) Visualization of the binding model of compound KZL-047 and protein-level prediction for RecQL1, WRN, RecQL4, and RecQL5; (**b**) Effect of SA on RecQ helicase mRNA expression levels in L-02 cells via RT-qPCR detection; (**c**) Effect of KZL-047 on SA-induced decrease in RecQ helicase mRNA expression in L-02 cells; (**d**) Effect of SA on RecQ helicase protein expression levels in L-02 cells; (**e**) Use GAPDH as a housekeeper gene. The effect of relative protein levels was quantified using Image J software (V1.8.0.112, NIH, Bethesda, MD USA); (**f**) Effect of KZL-047 on the expression of RecQ helicase proteins in SA-treated L-02 cells; (**g**) Statistical chart showing the effect of KZL-047 on RecQ helicase protein levels in L-02 cells treated with SA. Data are presented as mean ± SD of three independent experiments. * *p* < 0.05, ** *p* < 0.01, *** *p* < 0.001 compared with the control group; ^a^ *p* < 0.05, ^aa^ *p* < 0.01, ^aaa^ *p* < 0.001 compared with the model group (*n* = 3).

KZL-047 was docked with four target proteins of the RecQ family helicases: RecQL1, WRN, RecQL4, and RecQL5. The results showed that KZL-047 bound well to the four proteins (Table 1), and the most tightly bound was WRN, with a minimum Gibbs free energy of −8.2 kcal/mol. Pymol and Ligplus were used to visualize the docking results for KZL-047 with the four target proteins (Figure 4a). RT-qPCR was used to detect the effect of SA on RecQ helicase mRNA expression in L-02 cells. As shown in Figure 4b, compared with the control group, the model groups with SA concentrations of 20 and 40 μmol/L showed significantly different concentrations of the four target proteins (*p* < 0.01). Among them, the expression of RecQL1 mRNA increased with SA concentration first and then decreased, whereas the expressions of WRN, RecQL4, and RecQL5 mRNAs were negatively correlated with SA concentration. This result suggests that SA induced a decrease in RecQ helicase mRNA expression in L-02 cells. The effect of KZL-047 on SA-induced RecQ helicase mRNA expression changes in L-02 cells was also detected by RT-qPCR. As shown in Figure 4c, the expression levels of RecQL1, WRN, RecQL4, and RecQL5 mRNAs in L-02 cells decreased after treatment with 20 μmol/L SA (*p* < 0.05) compared with the control; after 5 and 10 μmol/L KZL-047 intervention, the expression levels of the abovementioned mRNAs significantly increased (*p* < 0.05) in a dose-dependent manner. This result suggests that KZL-047 reversed the decrease in RecQ helicase expressions induced by SA in L-02 cells.

### 2.3. Effect of KZL-047 on the RecQ Helicase Proteins Expression of L-02 Cells Analyzed by Western Blotting

The effect of SA and KZL-047 on the expression of RecQ helicase in L-02 cells was detected by Western blotting. As shown in Figure 4d,e, the expression levels of RecQL1, WRN, RecQL4, and RecQL5 decreased with an increase in SA concentration in a dose-dependent manner in L-02 cells for 24 h (*p* < 0.01). As shown in Figure 3f,g, the expressions of RecQL1, WRN, RecQL4, and RecQL5 in the model group were significantly reduced compared with the control group (*p* < 0.01); after intervention with 5 and 10 μmol/L KZL-047, the abovementioned RecQ helicase protein expressions increased in a dose-dependent manner, and the difference was statistically significant (*p* < 0.05) except for the difference in RecQL5 expression following treatment with 5 μmol/L KZL-047. This suggests that SA induced a decrease in RecQ helicase protein expression in L-02 cells, and KZL-047 reversed the decrease in RecQ helicase expressions induced by SA.

### 2.4. Effect of KZL-047 on SA-induced Liver Injury in Mice

#### 2.4.1. Effects of KZL-047 on Body Weight, Liver Weight, and Liver Coefficient in Mice

Prevention experiment group: As shown in Table 2, and compared with the control group, the model group showed significantly decreased body weight (*p* < 0.01) and increased liver weight and liver coefficient (*p* < 0.01). No significant differences were observed between the positive control group (Bicyclol, glutathione) and the KZL-047 dose groups. Compared with the model group, the intervention groups showed contrasting results for the abovementioned indicators, and there was no difference between the KZL-047 dose groups and the positive control group. Treatment experiment group: The trends concerning weight, liver weight, and liver coefficient for mice were consistent with those observed in the prevention experiment group. With an increase in KZL-047 dose, the body weight of mice increased (*p* < 0.01), whereas the liver weight and liver coefficient decreased (*p* < 0.01) in a dose-dependent manner.

#### 2.4.2. Effect of KZL-047 on Serum ALT, AST, and TBIL in Mice

Prevention experiment group: As shown in Table 3, compared with the blank group, the model group (Bicyclol, glutathione) showed significantly increased activities of ALT, AST, and TBIL (*p* < 0.01), and there were no significant differences between the positive control group and KZL-047 dose groups. Compared with the model group, the intervention groups showed significantly decreased activities of the abovementioned indicators (*p* < 0.01), and there were no differences between the KZL-047 dose groups and the positive control group. Treatment experiment group: The changes in serum ALT, AST, and TBIL activities in mice were similar to those observed in the prevention groups, and the changes in KZL-047 dose groups were dose-dependent.

#### 2.4.3. Effects of KZL-047 on Malondialdehyde (MDA), Glutathione (GSH), and Superoxide Dismutase (SOD) in Mouse Liver Tissue

Prevention experiment group: As shown in Table 4, compared with the control group, the model group showed significantly increased mouse liver MDA content (*p* < 0.01), and significantly decreased GSH content and SOD activity (*p* < 0.01). There were no significant differences between the positive control group (Bicyclol, glutathione) and KZL-047 dose groups; compared with the model group, the KZL-047 dose groups showed contrasting results for the abovementioned indicators, and there were no differences between the KZL-047 dose groups and the positive control group. Treatment experiment group: The changes in the results of MDA, GSH, and SOD in mouse liver tissues were consistent with those observed in the prevention experiment group. With an increase in the KZL-047 dose, MDA gradually decreased (*p* < 0.01), whereas GSH and SOD increased in a dose-dependent manner (*p* < 0.01).

#### 2.4.4. Effect of KZL-047 on Pathological Changes in Liver Tissues of Mice

**Figure 5:**
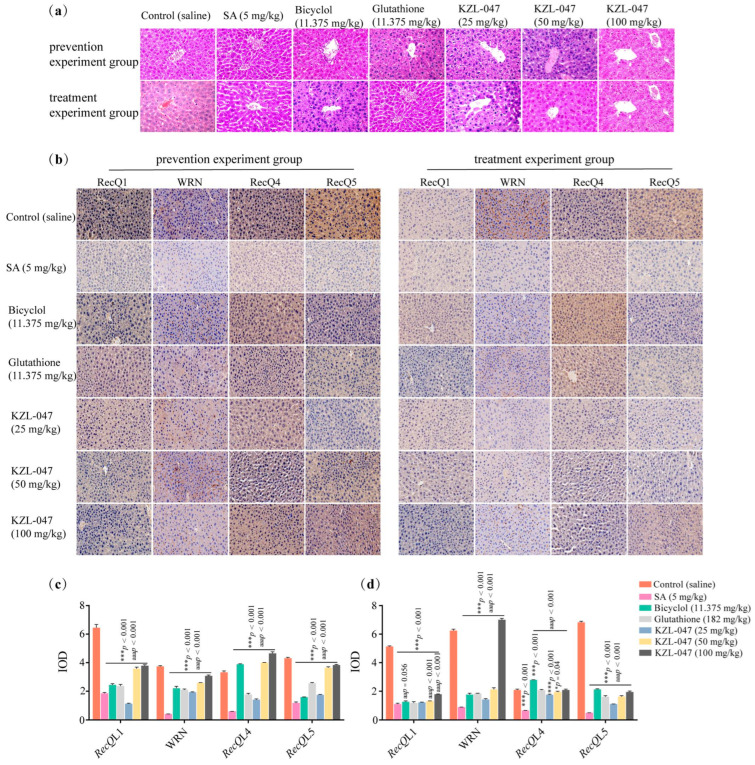
Effect of KZL-047 on pathological changes in liver tissues in mice. (**a**) Micrographs of pathological changes in liver tissues of mice in the prevention and treatment experiment groups (the magnification = ×400). (**b**) Immunohistochemical staining observation to prevent the expression of RecQ helicase in liver tissues of mice in the prevention and treatment experiment groups (the magnification = ×400). (**c**) Statistical results for changes in liver RecQ helicase in mice in each group under KZL-047 preventive treatment with SA. (**d**) Statistical results for changes in liver RecQ helicase in mice in each group treated with KZL-047 after SA exposure to mice. Data are expressed as mean ± SD of three independent experiments. * *p* < 0.05, *** *p* < 0.001, compared with the control group; ^aa^ *p* < 0.01, ^aaa^ *p* < 0.001 compared with the SA (5 mg/kg) group (*n* = 10).

Hematoxylin and eosin (H&E) staining showed pathological changes in the liver tissues of mice in the preventive experiment group. The morphology of hepatocytes in the liver tissue of mice in the control group was normal, the structure of the liver lobule was clear, and the central vein of the liver tissue was clear, without pathological changes; in contrast, in the arsenic-stained model group (SA 5 mg/kg), the liver lobule structure in the liver tissue of mice was unclear, the hepatic sinusoid was dilated, the interstitium had inflammatory cell infiltration, and the cell vacuoles were obvious. Inflammatory cells were occasionally seen in the liver tissues of mice in the positive control group; the pathological changes described above in the intervention group gradually improved with the increase in the dose of KZL-047. The overall structure of the liver tissues of the mice treated with 100 mg/kg of KZL-047 tended to be close to that of the control group. However, the pathological changes in the liver tissue of mice in the treatment experimental group were absent in the micrographs of the liver tissue of control mice, whereas in the model group (SA 5 mg/kg) the liver sinusoids were dilated and vacuoles were evident. The number of inflammatory cells was reduced in the positive control group; in the KZL-047-treated group, the structure of the liver lobules was clear, the hepatic cords were arranged in an orderly manner, and the infiltration of inflammatory cells was significantly reduced with the increase in the dose of KZL-047 (Figure 5a). The abovementioned results showed that KZL-047 has a certain alleviation effect on the prevention and treatment of SA-induced liver injury in mice, based on analyzing the changing trend in liver coefficient, serum liver function, and liver oxidative damage indicators. Keeping in mind the in vitro experimental results, KZL-047 was used to study the molecular mechanisms of arsenic-stained mice in vivo.

Changes in the expression of RecQ helicase in the liver tissues of mice in the preventive experiment group were seen under a light microscope, and RecQL1-, WRN-, RecQL4-, and RecQL5-positive cells were seen in all groups of mice and the control group. Blue nucleus, yellowish-brown cytoplasm, and intercellular spaces were observed, as shown in Figure 5b. In the control group, the positive cells were obvious with rich brown-yellow reaction products; the number of positive cells in the model group (SA 5 mg/kg) decreased significantly, indicating a decrease in the expression of the abovementioned genes (*p* < 0.01), and compared with the model group, the positive control group and KZL-047 groups showed significantly increased expression levels of these genes (*p* < 0.01). Changes in the expression of RecQ helicases in the liver tissues of mice in the treatment experiment groups were observed with a decrease in the number of gene-positive cells in the model group compared with the blank group (*p* < 0.01); the expression levels of positive cells in the livers of mice in the positive control group and KZL-047 groups increased (*p* < 0.01). The abovementioned findings suggest that KZL-047 prophylactic administration reduced the expression of RecQ helicases in mouse liver, and KZL-047 therapeutic administration increased the expression of RecQ helicases in mouse liver (Figure 5c,d).

## 3. Discussion

Arsenite can react with oxygen, chlorine, and sulfur in the environment to produce toxic soluble compounds that enter the human body through drinking water and diet [18]. It is estimated that nearly 108 countries are affected by arsenic contamination in groundwater, which indicates that it is a serious threat to human health [19]. Animal experimental studies have shown that arsenic accumulation in the body can cause different degrees of liver injury and even the development of tumors [20]. In recent years, more about the metabolic patterns of arsenite activity in vivo has been understood, including gene mutations, chromosomal aberrations, signal transduction, cell cycle regulation, apoptosis, and oxidative stress [21]. However, the basic mechanisms associated with these effects have not been verified, and there is a lack of effective drugs and clinical measures to treat arsenic-related diseases. To elucidate the molecular mechanisms of arsenic, cell models and animal models of arsenic have been established to advance the realization of effective therapeutic drugs [22].

Our study showed that arsenic induced apoptosis in L-02 cells, blocked cell cycle development, and decreased the mRNA and protein expression levels of RecQL1, WRN, RecQL4, and RecQL5 genes, which led to a decrease in the viability of L-02 cells after arsenic exposure. The degree of cell damage was positively correlated with arsenic exposure dose [23]. The inhibition of DNA damage and DNA repair following injury was considered to be the initial factor in the pathogenic and carcinogenic effects of arsenic exposure. Human RecQ helicase, as a DNA repair protein, plays an important role in DNA repair following injury [24]. KZL-047 was used for intervention in the arsenic-poisoning cell model, and the results showed that KZL-047 could effectively reverse the abovementioned changes induced by arsenic. Therefore, it is speculated that the changed DNA damage repair proteins after arsenic exposure may participate in cell apoptosis and cell cycle development by affecting the expression of RecQ helicase, leading to the occurrence of arsenic toxicity, which also provides a new entry point for further research on the mechanism of arsenic-induced toxicity of RecQ helicase.

Relevant studies have reported quinazoline derivatives to have varying degrees of mitigating effects on liver injury [25,26]. In the activity screening of pre-experimental compounds, we unexpectedly found that quinazoline derivative KZL-047 was able to promote the proliferation of L-02 cells. To verify whether it has an alleviating effect on arsenic-induced liver injury and to determine its molecular mechanism, arsenic-induced liver injury models were developed in vitro and in vivo to explore the prevention and treatment effect of the natural product KZL-047. In this study, a mouse liver injury model was successfully constructed using 5 mg/kg SA gavage in mice. The body weight of mice in the model group decreased and the liver coefficient increased. In hepatocytes, ALT mainly exists in the cytoplasm, whereas approximately 80% of AST exists in the mitochondria [27]. When hepatocytes are damaged, the permeability of the cell membrane increases, and the ALT and AST in the cytoplasm are released into the blood, increasing the activity of ALT and AST enzymes in the serum [28]. Bicyclol and glutathione are commonly used drugs to treat hepatitis and have important roles such as antioxidation and scavenging of free radicals in the body. The former can significantly reduce the degree of elevation of serum ALT and AST and the degree of liver tissue necrosis in mice with liver injury, maintain cell stability, and play a role in protecting the liver [29]. The latter can effectively improve the liver injury caused by oxidative stress reactions as a result of GSH deficiency in the body [30]. The results of this study showed that after KZL-047 intervention, the ALT and AST levels in mice significantly decreased in a concentration-dependent manner, indicating that KZL-047 could improve the hepatic disorders induced by SA.

Arsenic can make the body produce more reactive oxygen species, inhibit the activity of antioxidants, reduce the antioxidant capacity, and lead to oxidative damage to the body [25]. MDA is a lipid peroxidation product, SOD and GSH act as important scavengers of superoxide radicals in the body.Changes in the levels of MDA, SOD and GSH can reflect the degree of oxidative stress in the body [26]. The results of this study showed that the liver tissues of mice in the model group showed a significant increase in MDA content and decrease in GSH and SOD activity, and KZL-047 was able to reverse the levels of oxidative stress indicators (MDA, GSH and SOD), suggesting that KZL-047 can effectively scavenge oxygen radicals, enhance the body’s antioxidant capacity and inhibit hepatic lipid peroxidation, thus further alleviating arsenic-induced liver injury.

Histopathological studies have found that arsenic-induced liver toxicity is manifested by varying degrees of hepatocellular necrosis, fibrosis, or hyperplasia, accompanied by large amounts of collagen deposition and hepatic stellate cell activation [31]. In the control group, the structure of liver lobules was intact and clear, the liver parenchymal cells showed no obvious lesions; there was no inflammatory cell infiltration in liver tissues; in contrast, in the model group, the hepatocytes of the mice were disorganized and there was necrosis of some hepatocytes. For the abovementioned pathological changes, the liver tissues of mice in the Bicyclol, glutathione, and KZL-047 groups showed different degrees of remission. Immunohistochemical staining of mouse liver tissues showed that the expression levels of RecQL1, WRN, RecQL4, and RecQL5 were significantly decreased in mouse liver tissues after KZL-047 intervention compared with those before intervention. This finding was consistent with the results of the in vitro study. Our histological results support discoveries at the biochemical and molecular levels. The course of liver injury caused by the environmental pollutant arsenic is long, and clinical symptoms do not easily appear in a short time, which brings certain difficulties to its diagnosis and treatment [32]. Thus, it is particularly important to prevent the occurrence and development of arsenic toxicity.

The results of this study showed that the improvement in liver biochemical indicators in the prevention group was more obvious than that in the treatment group, suggesting that the preventive effect of KZL-047 was better than the treatment effect. In vivo and in vitro studies have shown that KZL-047 was able to promote the proliferation of sodium arsenite-induced injury L-02 cells, alleviate sodium arsenite-induced apoptosis of L-02 cells, and attenuate the sodium arsenite-induced G1-phase blockade of L-02 cells, and, at the same time, it was able to reduce the liver function (ALT, AST, TBIL) and oxidative stress levels (MDA, GSH, SOD) in arsenic-poisoned mice, which could attenuate the arsenic poisoning symptoms in mice. The related mechanism may be related to the role of KZL-047 in reversing sodium arsenite-induced RecQ helicase protein expression in L-02 cells. Further experiments are needed to clarify the mechanism of this protective function in the future.

## 4. Materials and Methods

### 4.1. Materials, Cells, and Experimental Animals

All types of equipment and biological agents used in this study were as previously reported [33]. Normal human hepatocyte L-02 cell line was purchased from the Cell Resource Center of Shanghai Academy of Biological Sciences, Chinese Academy of Sciences, and stored in the Key Laboratory of Chemistry for Natural Products of Guizhou Province and Chinese Academy of Sciences (Guiyang, China).

Compound KZL-047 was synthesized according to a previously reported procedure [16,17] using 2-amino-4,5-dimethoxybenzamide as the starting material, and the target compound was obtained as a yellow solid. NMR parameters were as follows: ^1^H NMR (600 MHz, DMSO-*d_6_*) *δ* 9.83 (s, 1H), 7.94 (s, 1H), 7.67 (d, *J* = 4.4 Hz, 2H), 7.35 (s, 1H), 7.01 (d, *J* = 4.1 Hz, 2H), 3.98 (s, 3H), 3.96 (s, 3H), 3.79 (s, 3H), 2.50 (s, 3H); ^13^C NMR (151 MHz, DMSO-*d_6_*) *δ* 157.63, 156.53, 155.28, 150.72, 150.26 (q, *J* = 34.0 Hz), 146.25, 131.95, 124.67, 123.36, 121.53, 120.66 (q, *J* = 263.1 Hz), 119.70, 118.04, 114.23, 109.37, 108.30, 102.57, 56.84, 56.54, 55.70; and ^19^F NMR (565 MHz, DMSO-*d_6_*) *δ* −69.16 (s).

The experimental animals used in this study were 140 SPF male Kunming mice, aged 8–10 weeks, weighing 25–30 g. The mice were provided by Tengxinbier Experimental Animal Sales Co., Ltd. (Chongqing, China), with an animal production license (Certificate No.: SCXK (Liao) 2020-0001), and all animal experiments were approved by the experimental animal ethics committee (No. 2101015). The mice were raised in the SPF animal room of the Experimental Animal Center of Guizhou Medical University in the barrier environment of the IVC system. The certificate number of the experimental facility is SYXK (Qian) 2018-0001. The temperature set for raising the mice was 22 ± 2 °C, and the humidity was 50 ± 10%. The mice were housed in a normal light–dark cycle and had food and water ad libitum. The feed and bedding for mice were SPF grade, and the drinking water provided had been distilled after high-temperature and high-pressure sterilization.

### 4.2. Cell Culture and Compound Treatment

The L-02 cells were grown in DMEM (Gibco, Waltham, MA, USA) supplemented with 10% fetal bovine serum (FBS) and 1% penicillin/streptomycin (Solarbio, Beijing, China) at 37 °C with 5% CO_2_. The L-02 cell line was characterized by STR analysis and confirmed not to contain mycoplasma contamination. The selected compound KZL-047 is a quinazoline derivative, with a purity of more than 99%. KZL-047 was solubilized as a 2 × 10^4^ μmol/L solution using DMSO and stored at −20 °C.

### 4.3. Cell Growth Assay

#### 4.3.1. Cell Proliferation Assay

The effect of SA on L-02 cell proliferation was determined by an MTT colorimetric assay. In brief, L-02 cells (5 × 10^3^ cells/well) were seeded into 96-well plates with 190 μL DMEM, supplemented with 10% fetal bovine serum, and incubated overnight at 37 °C with 5% CO_2_. The experimental group was treated with graded concentrations of SA (10, 20, 40, 80, and 160 μmol/L), the control group was treated with 0.1% DMSO for different durations (12, 24, and 48 h), and the number and morphology of the cells were observed by inverted fluorescence microscopy (Nikon, Japan). Each sample had five replicate wells. In the experiment, 20 μL MTT (5 mg/mL) was added to each well followed by incubation for 4 h. The supernatant was discarded by centrifugation and 150 μL DMSO was added and shaken for 15 min to dissolve the Formazan crystals. The absorbance was spectrophotometrically measured at 490 nm using a microplate reader (BioTek, Winooski, VE, USA).

#### 4.3.2. EdU (5-ethynyl-2’-deoxyuridine) Assay

The effect of SA on DNA synthesis in the cells was evaluated via (EDU)-DNA synthesis assay using the EdU Apollo567 in vitro kit (Solarbio, Beijing, China) [34]. First, 1 × 10^4^ cells per well were seeded in 96-well plates and incubated for 24 h. Next, SA was added at different concentrations and cells were incubated for an additional 48 h. Briefly, 100 μL of 50 µmol/L EdU medium was added to each well and after 2 h of incubation, the medium was discarded and the cells were rinsed 3 times with 100 μL of PBS for 5 min each time. Each well was incubated with 50 μL of cell fixative (PBS containing 4% paraformaldehyde) for 30 min at room temperature, the fixative was discarded, and the 96-well plate was shaken with 50 μL of 2 mg/mL glycine on a shaker for 5 min and then rinsed with PBS. Then, 100 μL of permeabilizer (0.5% Trixon-X-100 in PBS) was added to each well, incubated for 10 min, and rinsed with PBS. The wells were incubated with 1× Apollo Staining Reaction Solution for 30 min at room temperature and rinsed with PBS, and then stained with 1× Hoechst 33342 Reaction Solution for 30 min at room temperature and protected from light. Each well was washed 3 times with PBS. Finally, the stained cells were observed under a fluorescence microscope (Leica, Wetzlar, Germany).

#### 4.3.3. Hoechst 33258 Staining

The effect of KZL-047 on the apoptosis of L-02 cells was detected by Hoechst 33258 staining. Washed coverslips were put into 6-well plates, inoculated with an appropriate number of cells, and put into the incubator to continue incubation until 70% confluency, following which the old culture medium was drained. Hoechst 33258 (1 mg/mL) staining solution was added, and cells were stained for 5 min at room temperature in the dark. The morphological changes in cell nuclei were observed under an inverted fluorescence microscope to determine apoptosis.

#### 4.3.4. Mitochondrial Membrane Potential (MMP) Assay

The effect of KZL-047 on the apoptosis of L-02 cells was detected by JC-1 cell membrane potential assay. The cells were placed under a fluorescence microscope to observe the color change in cells and photographed.

#### 4.3.5. Cell Apoptosis Assay

The effects of KZL-047 on L-02 cell apoptosis were detected by flow cytometry using an Annexin V-fluorescein isothiocyanate (FITC) and propidium iodide (PI) staining kit (BD Pharmingen, San Diego, CA, USA) [35]. In all, 5 × 10^5^ L-02 cells were seeded in 6-well plates and treated with different concentrations (10, 20, 40 μmol/L) of SA for 24 h. DMSO 0.1% was used as the control. Next, the cells were digested, centrifuged, and washed with PBS. Cells were incubated with 5 μL Annexin V-FITC and 5 μL PI for 15 min, and a single incubation group was set to adjust fluorescence compensation. Flow cytometry (BD Biosciences, San Jose, CA, USA) was performed to quantitatively detect the apoptosis rate of cells treated with different concentrations of SA [33].

#### 4.3.6. Cell Cycle Assay

The effects of KZL-047 on the L-02 cell cycle were detected by flow cytometry. The procedure to inoculate and treat the cells as described in Section 4.3.5. Cells were fixed in 70% ice-cold ethanol for 4 h or even longer at 4 °C [36]. The cells were then centrifuged and collected, followed by adding the cells to a 25 μL RNAse water bath for 30 min. The cells then underwent PI photophobic dyeing (5 μL) for 15 min. Flow cytometry was performed to quantitatively detect the effects of treatment with different concentrations of SA on the cell cycle.

### 4.4. Gene and Protein Expression Assay

#### 4.4.1. Molecular Docking Assay

The structure of KZL-047 was drawn using ChemDraw 20.0 software (PerKinelmer Instruments Co., Ltd., Waltham, MA, USA) and saved in mol.2 format. The three-dimensional coordinates of RecQ helicase were taken from the protein database (PDB ID:3ZS5 and 3GHW). After decompression, the “remove solvent, remove organic” programming operation was performed, and the optimized target protein and KZL-047 were uploaded to the Swissdock website (http://www.swissdock.ch/, accessed on 22 May 2022) to perform molecular docking. Open Babel was used for preprocessing to facilitate structure-based molecular docking in AutoDock for visualization purposes. In addition to visualization, these tools can be used to measure the bond length, the distance between two coordinates, and the distance between nucleotides and ligands. Online resources such as PubChem and PDB SwissADME were used for retrieval, evaluation, and analysis to provide an intuitive understanding of binding sites and binding residues at a molecular level.

#### 4.4.2. RNA Extraction and Reverse Transcription Assay

To determine the regulatory role of RecQ family DNA helicases at an mRNA level, the RNA was isolated from L-02 cells treated with different concentrations of SA using the total RNA extraction reagent TRIzol (Thermo Fisher Scientific, Waltham, MA, USA). Cells were collected and lysed, and centrifuged at 12,000 rpm and 4 °C for 15 min. Chloroform was then added to separate the mixture by establishing a transparent upper aqueous layer containing RNA, followed by precipitation with isopropyl alcohol and washing with 75% ethanol. These steps were performed on ice. Concentration and purity were determined using a micro nuclein quantitative instrument, NanoDrop 2000 (Thermo Fisher Scientific, Waltham, MA, USA), purchased from Thermo Fisher Scientific Technologies. A260/A280 samples in the range 1.82.0 were retrotranscribed into cDNA using the HiFiScript cDNA synthesis kit (CW Bio, Beijing, China).

#### 4.4.3. Real-Time Fluorescence Quantitative PCR Assay

The gene sequences of *RecQL1*, *WRN*, *RecQL4,* and *RecQL5* were obtained from the primer gene bank NCBI according to the primer design requirements. *GAPDH* was used as the experimental reference (Beijing Qingke Biotechnology Co., Ltd., Beijing, China) to perform real-time quantitative RT-PCR (qRT-PCR) for the primers of human RecQ helicase. The UItraSYBR Green qPCR Mixture (ROX-contained) kit (Beijing Kangwei Century Biotechnology Co., Ltd.) was used to configure the reaction system under dark conditions, and the expression of RecQ helicases was detected in the Plus™ real-time fluorescence PCR system (Applied Biosystems, Waltham, MA, USA) after shaking and mixing [33]. The reaction conditions were as follows: initial denaturation at 95 °C for 10 min, followed by 40 cycles at 95 °C for 15 s, and 60 °C for 1 min. The 2^−ΔΔCT^ method was used to calculate the relative expression levels. The primer sequences are listed in Table 5.

#### 4.4.4. Western Blotting

L-02 cells treated with different concentrations of SA for 24 h were collected, lysed on ice for 30 min, and centrifuged at 12,000 rpm/min for 15 min at 4 °C; the proteins were subsequently collected. The protein concentration was determined by BCA protein assay. Proteins (50 µg/per line) were separated by 10% SDS-PAGE, and total proteins were transferred to a polyvinylidene difluoride (PVDF) membrane. The membranes were then incubated overnight at 4 °C with the following primary antibodies: BLM (1:1000), WRN (1:1000), RECQL4 (1:1000), and RECQL5 (1:1000) (Abcam, UK). The membranes were washed with TBST and then incubated with goat antirabbit IgG H+L secondary antibody (1:30,000). GAPDH was used as a control. Immunoreactive proteins were detected using the Odyssey infrared imaging system (Gene Company Limited, LI-COR, HK, China), and protein expression levels of the relevant genes were measured by banded grayscale values using Image J software (V1.8.0.112, NIH, Bethesda, MD, USA) [35].

### 4.5. Animal Experiment

#### 4.5.1. Grouping and Processing

In all, 140 SPF male Kunming mice were acclimatized to feeding for 1 week before formal experiments. Mice were randomly divided into the prevention and treatment experiment groups according to their weight. Mice were administered drugs by gavage with 0.1 mL/g body weight. The control group (negative control) was administered normal saline; Bicyclol (11.375 mg/kg) and glutathione (182 mg/kg), commonly used in clinical liver protection treatment, were used as positive controls. The mouse liver injury model was established by administration of 5 mg/kg SA according a protocol reported in the literature [37]. Based on the results of Huailing Wei et al., the dose design of KZL-047 was 25, 50, and 100 mg/kg. KZL-047 and positive control drugs were given 30 min before intragastric administration of SA to mice in the prevention experiment group for 28 days. The mice in the treatment experiment group were given KZL-047 and positive control drugs every day for 28 days after SA exposure.

After the last administration, mice were fasted from food and water. Mouse body weight was measured and recorded the next morning. Mice were anesthetized with isoflurane and euthanized by cardiac puncture. Blood samples were collected from mice by the orbital venous plexus blood collection method. After standing the blood samples at room temperature for 30 min, the supernatant as the serum sample was collected by centrifugation at 3000 rpm for 15 min, and frozen in a refrigerator at −80 °C for subsequent experiments.

#### 4.5.2. Liver Coefficient Assay

The mice were weighed before sacrificing them, the liver was weighed after dissection, and the liver coefficient was calculated as follows: liver coefficient = (liver weight/body weight before death) × 100%.

#### 4.5.3. Biochemical Indicators in Serum Assay

The activity of alanine transaminase (ALT) and aspartate aminotransferase (AST) in the serum was determined by the rate method, and the total bilirubin (TBIL) level was determined by the two-point method. An automatic biochemical analyzer (Shenzhen Leidu Life Science and Technology Co., Ltd., Shenzhen, China) was used for calibration. The specific operation was performed according to the kit instructions.

#### 4.5.4. Indicators of Oxidative Stress in Liver Tissue Assay

An appropriate amount of liver tissue was cut, precooled normal saline (tissue (g):normal saline (mL) = 1:9) was added, and a 0.1 mL 10% liver tissue homogenate was prepared using a high-speed and low-temperature tissue grinder. The content of malondialdehyde (MDA) was determined by the thiobarbituric acid (TBA) method, the activity of superoxide dismutase (SOD) was determined by the WST-1 method, and the content of glutathione peroxidase (GSH) was determined by the microplate method. All indicators were tested in strict accordance with the instructions of the corresponding kits, and a microplate reader was used for detection.

#### 4.5.5. Pathological Changes in Liver Tissue Assay

An appropriate amount of liver tissue sample was fixed in 4% paraformaldehyde and cut into small pieces of uniform thickness with a scalpel [38]. The liver tissues were dehydrated, dipped in wax, embedded, stained, and examined under a microscope, and the pathomorphological changes were observed and photographed under a microscope.

#### 4.5.6. Immunohistochemical Assay

The liver paraffin sections were dewaxed by the gradient method, and antigen retrieval and endogenous peroxidase blockade were performed in a microwave oven [39]. After the slices were incubated overnight at 4 °C with the first antibody, the second antibody corresponding to the first antibody was dropped; DAB was used for color development. Water was used to wash and stop dyeing; hematoxylin was used for dyeing, and neutral resin was used to mount the tablets. A light microscope was used to observe and take photographs. The Image J image processing system was used to observe the expression levels of RecQL1, WRN, RecQL4, and RecQL5. A brownish-yellow color indicated positive expression, and the integral optical density (IOD) of liver tissue staining was tested.

### 4.6. Statistical Analysis

Statistical analysis was performed using IBM SPSS Statistics 21.0 and GraphPad Prism 7.0 software. Data are presented as $\overset{-}{x}$ ± *s* of three independent experiments. The Student *t*-test was used to evaluate the significance between the two groups, whereas multiple-group comparisons were evaluated by two-way ANOVA with Tukey’s test for post hoc testing. The presented results are representative of three independent experiments and *p* < 0.05 was considered to indicate statistical significance.

### 4.7. Patents

The manuscript contains a patent arising from patent application NO. CN202210084671.5.

## Figures and Tables

**Table 1:** Molecular docking results for four key target proteins.

Target Symbol	Entry	PDB ID	Binding Affinity (kcal/mol)
RecQL1	P46063	2V1X	−7.9
WRN	Q14191	6YHR	−8.2
RecQL4	O94761	5LST	−6.6
RecQL5	O94762	5LB3	−7.5

**Table 2:** Changes in body weight, liver weight, and liver coefficient for mice (*x* ± *s*, *n* = 10).

Group (mg/kg)	Prevention Experiment Group			Treatment Experiment Group		
	Body Weight, g	Liver Weight, g	Liver Coefficient, %	Body Weight, g	Liver Weight, g	Liver Coefficient, %
Control (saline)	47.40 ± 0.93	1.78 ± 0.08	3.75 ± 0.22	51.6 ± 1.11	1.91 ± 0.06	3.68 ± 0.12
SA (5.0)	35.07 ± 1.77 **	2.20 ± 0.06 **	6.27 ± 0.35 ^aa^	43.83 ± 1.78 **	2.31 ± 0.05 **	5.27 ± 0.30 **
Bicyclol (11.375)	46.13 ± 1.16 ^aa^	1.89 ± 0.12 ^aa^	4.10 ± 0.23 ^aa^	50.05 ± 1.34 ^aa^	1.90 ± 0.06 ^aa^	3.79 ±0.20 ^aa^
glutathione (182)	47.43 ± 1.29 ^aa^	1.87 ± 0.17 ^aa^	3.94 ± 0.40 ^aa^	50.92 ± 0.92 ^aa^	1.95 ± 0.04 ^aa^	3.83 ± 0.07 ^aa^
KZL-047 (25)	45.50 ± 1.90 ^aa^	1.85 ± 0.08 ^aa^	4.08 ± 0.18 ^aa^	50.07 ± 1.47 ^aa^	1.99 ± 0.10 ^aa^	3.97 ± 0.10 ^aa^
KZL-047 (50)	46.18 ± 1.68 ^aa^	1.86 ± 0.05 ^aa^	4.03 ± 0.17 ^aa^	50.98 ± 1.24 ^aa^	1.90 ± 0.05 ^aa^	3.74 ± 0.12 ^aa^
KZL-047 (100)	47.35 ± 1.54 ^aa^	1.78 ± 0.11 ^aa^	3.76 ± 0.18 ^aa^	51.17 ± 1.27 ^aa^	1.89 ± 0.05 ^aa^	3.69 ± 0.06 ^aa^

** *p* < 0.01, compared with the control group; ^aa^ *p* < 0.01, compared with the model group.

**Table 3:** Changes in serum ALT, AST, and TBIL in mice (*x* ± *s*, *n* = 10).

Group (mg/kg)	Prevention Experiment Group			Treatment Experiment Group		
	ALT, U/mL	AST, U/mL	TBIL, μmol/L	ALT, U/mL	AST, U/mL	TBIL, μmol/L
Control (saline)	35.01 ± 6.37	99.08 ± 5.45	19.20 ± 4.99	45.54 ± 4.10	78.95 ± 5.36	15.36 ± 1.26
SA (5.0)	71.59 ± 11.75 **	174.24 ± 26.66 **	37.56 ± 7.90 **	86.60 ± 10.62 **	154.30 ± 14.26 **	32.04 ± 4.73 **
Bicyclol (11.375)	36.98 ± 4.74 ^aa^	100.12 ± 2.67 ^aa^	18.25 ± 4.52 ^aa^	52.48 ± 9.67 ^aa^	77.40 ± 2.91 ^aa^	16.39 ± 2.30 ^aa^
glutathione (182)	34.81 ± 3.91 ^aa^	97.42 ± 2.44 ^aa^	17.22 ± 4.37 ^aa^	43.84 ± 4.10 ^aa^	76.00 ± 3.38 ^aa^	15.09 ± 1.83 ^aa^
KZL-047 (25)	35.03 ± 0.96 ^aa^	96.93 ± 5.65 ^aa^	18.23 ± 4.24 ^aa^	52.36 ± 8.68 ^aa^	79.54 ± 5.98 ^aa^	14.98 ± 5.94 ^aa^
KZL-047 (50)	33.98 ± 0.93 ^aa^	94.78 ± 5.34 ^aa^	16.50 ±3.38 ^aa^	49.38 ± 5.23 ^aa^	78.02 ± 4.14 ^aa^	14.80 ± 2.82 ^aa^
KZL-047 (100)	32.76 ± 1.03 ^aa^	91.89 ± 8.68 ^aa^	15.12 ± 2.27 ^aa^	44.28 ± 3.05 ^aa^	75.23 ± 3.88 ^aa^	14.28 ± 3.36 ^aa^

** *p* < 0.01, compared with the control group; ^aa^ *p* < 0.01, compared with the model group.

**Table 4:** Changes in MDA, GSH, and SOD in liver tissues of mice (*x* ± *s*, *n* = 10).

Group (mg/kg)	Prevention Experiment Group			Treatment Experiment Group		
	MDA nmol/mg	GSH, μmol/g	SOD, U/g	MDA, nmol/mg	GSH, μmol/g	SOD, U/g
Control (saline)	25.69 ± 2.67	39.60 ± 1.89	151.12 ± 3.19	15.19 ± 0.59	37.16 ± 5.93	96.09 ± 8.34
SA (5.0)	36.71 ± 3.65 **	21.06 ± 1.42 **	95.97 ± 3.33 **	26.14 ± 4.14 **	21.41 ± 1.40 **	50.60 ± 7.76 **
Bicyclol (11.375	28.53 ± 3.15 ^aa^	37.79 ± 0.73 ^aa^	147.17 ± 3.39 ^aa^	16.83 ± 1.70 ^aa^	34.25 ± 0.51 ^aa^	87.24 ± 5.86 ^aa^
glutathione (182)	27.87 ± 4.10 ^aa^	38.19 ± 0.87 ^aa^	147.72 ± 3.54 ^aa^	16.07 ± 1.55 ^aa^	35.22 ± 1.04 ^aa^	88.84 ±3.16 ^aa^
KZL-047 (25)	26.05 ± 4.10 ^aa^	37.89 ± 0.79 ^aa^	145.62 ± 5.19 ^aa^	16.84 ± 2.53 ^aa^	35.07 ± 0.84 ^aa^	88.55 ± 1.66 ^aa^
KZL-047 (50)	25.80 ± 1.68 ^aa^	38.26 ± 1.75 ^aa^	147.75 ± 5.01 ^aa^	15.07 ± 1.14 ^aa^	39.02 ± 3.84 ^aa^	90.13 ±1.21 ^aa^
KZL-047 (100)	25.72 ± 0.43 ^aa^	38.56 ± 1.50 ^aa^	148.41 ± 6.09 ^aa^	14.85 ± 1.22 ^aa^	39.42 ± 3.62 ^aa^	74.13 ± 5.68 ^aa^

** *p* < 0.01, compared with the control group; ^aa^ *p* < 0.01, compared with the model group.

**Table 5:** The sequences of the primers used in this study.

Gene	Primer	Primer Sequence
RECQL1	Forward Reverse	5′-AAGAATGGCGTCCGTTTCAG-3′ 5′-AGCTCTTGTTGCCTTTCCGT-3′
WRN	Forward Reverse	5′-GGCACGGTAGAACCAACTCA-3′ 5′-TGCTCTTCATTGGGTGCTGG-3′
RECQL4	Forward Reverse	5′-ATCCTGTCTGGCATCTCCAC-3′ 5′-TGCAGGACAGATTCCCGTTG-3′
RECQL5	Forward Reverse	5′-AGCCACCATACCACCTTTCC-3′ 5′-GGGCATGCACACAAAGACG-3′
GAPDH	Forward Reverse	5′-GGAGTCCACTGGCTCTTCA-3′ 5′-GTCATGAGTCCTTCACGATACC-3′

## Data Availability

The data presented in this article are available.
